# Higher incidence of depression and anxiety in White females and those with a history of smoking one year after first-time hidradenitis suppurativa diagnosis: A retrospective cohort study

**DOI:** 10.1016/j.jdin.2025.05.005

**Published:** 2025-06-07

**Authors:** Cristo A. Carrasco Mendoza, Anna C. Davis, Emilie J. O’Banion, Stacy M. Castellanos, Zoe Q. Li, Jason R. Castillo

**Affiliations:** a Kaiser Permanente Bernard J. Tyson School of Medicine, Pasadena, California; b Department of Health Systems Science, Kaiser Permanente Bernard J. Tyson School of Medicine, Pasadena, California; c Center for Effectiveness and Safety Research, Kaiser Permanente National Research and Quality Measurement, Pasadena, California; d Office of Research and Scholarship, Kaiser Permanente Bernard J. Tyson School of Medicine, Pasadena, California; e Department of Research and Evaluation, Kaiser Permanente Southern California, Southern California Permanente Medical Group, Pasadena, California; f Department of Mohs Surgery, Barranca Medical Offices, Southern California Permanente Medical Group, Irvine, California

**Keywords:** anxiety, depression, epidemiology, ethnicity, hidradenitis suppurativa, incidence, race

*To the Editor:* Hidradenitis suppurativa (HS) is a chronic inflammatory skin disease marked by painful nodules in apocrine gland areas, and in later stages, abscesses, draining sinuses, and bridged scars.^1^ HS is commonly associated with comorbidities, especially depression and anxiety.^2^ This study aimed to assess the 1-year incidence of depression and anxiety following a first-time HS diagnosis. A secondary aim was to explore the association between adalimumab prescription and these mental health outcomes.

A retrospective cohort study was conducted using electronic health record data from Kaiser Permanente Southern California from 2010 to 2020. Eligible patients were those who (1) had a first-time HS diagnosis between January 1, 2011, and December 31, 2019; (2) were 18 years or older at the time of diagnosis; and (3) had continuous Kaiser Permanente Southern California membership for at least 1 year prior to their HS diagnosis (Supplementary File, available via Mendeley at https://data.mendeley.com/datasets/3wm8cz4n87/1).

Eligible patients were followed for 1 year after their first-time HS diagnosis (index date) to identify incident cases of depression and anxiety (Supplementary File, available via Mendeley at https://data.mendeley.com/datasets/3wm8cz4n87/1). Covariates included age, sex, race/ethnicity (White, Asian/Pacific Islander, Black, Hispanic, Native American/Alaskan Native/Multiple/Other, Unknown), body mass index (BMI), smoking status (ever, never), and comorbid conditions (diabetes, hypertension, hyperlipidemia). Additionally, adalimumab prescription after the index date was recorded. Data were analyzed using multivariate logistic regression.

A total of 3971 patients met study criteria (Table I). One year after the index date, 269 patients (6.8%) were diagnosed with depression and/or anxiety. Of these 86% were diagnosed with depression only and 30% with anxiety only. The mean age at first-time HS diagnosis was 38, with 74.5% of patients being female. The racial/ethnic breakdown of patients with a first-time HS diagnosis was as follows: Hispanic (40.3%), White (26.3%), Black (23.4%), and Asian/Pacific Islander (7.5%). The mean BMI was 34.

**Table I:** Characteristics of patients with first-time hidradenitis suppurativa diagnosis, overall, and by receipt of new diagnosis with depression or anxiety within 1 year after index date; 2010-2019

	Overall, *N* = 3971 (*n*)	Depression/anxiety diagnosis: no, *N* = 3702 (*n* (%))	Depression/anxiety diagnosis yes, *N* = 269	*P* value
Age at index date				.202∗
18-29	1476	1387 (94%)	89 (6.0%)	
30-39	962	882 (92%)	80 (8.3%)	
40-49	630	586 (93%)	44 (7.0%)	
50-59	504	470 (93%)	34 (6.7%)	
60+	399	377 (94%)	22 (5.5%)	
BMI				.121∗
Underweight/healthy	550	505 (92%)	45 (8.2%)	
Overweight	895	846 (95%)	49 (5.5%)	
Obese	2491	2318 (93%)	173 (6.9%)	
Unknown	35	33	2	
Sex				**.001**∗
Male	1012	966 (95%)	46 (4.5%)	
Female	2959	2736 (92%)	223 (7.5%)	
Race and ethnicity				**.043**†
White	1043	956 (92%)	87 (8.3%)	
Asian/Pacific Islander	296	284 (96%)	12 (4.1%)	
Black	928	876 (94%)	52 (5.6%)	
Hispanic	1599	1486 (93%)	113 (7.1%)	
Other	60	56 (93%)	4 (6.7%)	
Unknown	45	44	1	
Tobacco use				**<.001**∗
Never	2579	2432 (94%)	147 (5.7%)	
Yes	1356	1236 (91%)	120 (8.8%)	
Unknown	36	34	2	
Diabetes				.858∗
No	3439	3207 (93%)	232 (6.7%)	
Yes	532	495 (93%)	37 (7.0%)	
Hypertension				.972∗
No	3141	2928 (93%)	213 (6.8%)	
Yes	830	774 (93%)	56 (6.7%)	
Hyperlipidemia				.561∗
No	3155	2945 (93%)	210 (6.7%)	
Yes	816	757 (93%)	59 (7.2%)	

Bolded *P* values indicate statistical significance (*P* < .05).

*BMI*, Body mass index.

∗ Pearson’s chi-squared test.

† Fisher exact test.

In multivariate analysis, adjusting for age, sex, BMI, tobacco use, adalimumab prescription, and comorbidities, females had 94% higher odds of depression/anxiety than males (odds ratio [OR] = 1.94, *P* < .001) (Table II). Asian/Pacific Islanders (OR = 0.49, *P* = .025) and Black patients (OR = 0.65, *P* = .023) had lower odds compared to White patients. Tobacco use increased the odds of depression/anxiety by 75% (OR = 1.75, *P* < .001). Overweight individuals (BMI 25-29.9) had lower odds of depression/anxiety than those with normal/underweight (BMI <25) (OR = 0.65, *P* = .046). Adalimumab prescription (OR = 0.72, *P* = .331) was not significantly associated with our outcomes.

**Table II:** Logistic regression for 1-year incidence of depression or anxiety diagnosis among patients with first-time hidradenitis suppurativa diagnosis

	Adjusted∗		
	OR	95% CI	*P* value
Sex			
Male	—	—	
Female	1.94	1.38, 2.72	**<.001**
Race and ethnicity			
White	—	—	
Asian/Pacific Islander	0.49	0.26, 0.91	**.025**
Black	0.65	0.45, 0.94	**.023**
Hispanic	0.88	0.64, 1.20	.422
Other	0.60	0.18, 1.98	.403
Age at index date			
18-29	—	—	
30-39	1.37	0.99, 1.89	.057
40-49	1.05	0.70, 1.56	.823
50-59	0.96	0.59, 1.54	.854
60+	0.71	0.39, 1.28	.251
BMI			
Underweight/healthy	—	—	
Overweight	0.65	0.42, 0.99	**.046**
Obese	0.76	0.53, 1.10	.144
Tobacco use			
Never	—	—	
Yes	1.75	1.34, 2.30	**<.001**
Hypertension			
No	—	—	
Yes	1.07	0.72, 1.57	.750
Diabetes			
No	—	—	
Yes	1.02	0.67, 1.56	.928
Hyperlipidemia			
No	—	—	
Yes	1.22	0.84, 1.79	.294
Adalimumab ordered after index date			
No	—	—	
Yes	0.72	0.37, 1.39	.331

Bolded *P* values indicate statistical significance (*P* < .05).

*BMI*, Body mass index; *CI*, confidence interval; *OR*, odds ratio.

∗ Multivariate logistic regression.

To date, 2 studies have examined incident depression in patients with HS, with one of these also investigating incident anxiety.^3^^,^^4^ Our study highlights significant associations that support the utility of screening for depression and anxiety in patients newly diagnosed with HS, thereby informing mental health screening guidelines for this population.^5^

Our study had some limitations. Using diagnostic data for case ascertainment may exclude undiagnosed depression and anxiety cases. Second, our analysis was focused on patients with HS; it is not clear whether patterns in incidence of depression and anxiety that we observed in the HS population are different from those of the general population.

## Conflicts of interest

None disclosed.
